# Supplementary material for: Comprehensive Constitutional Genetic and Epigenetic Characterization of Lynch-Like Individuals
Source: Cancers (Basel). 2020 Jul 5;12(7):1799. doi: 10.3390/cancers12071799 (PMC7408773; doi:10.3390/cancers12071799)
Supplement: Supplementary file 1 [file cancers-12-01799-s001.zip › Figure S4_Pathogenicity assessment of MSH3 variants.pdf]

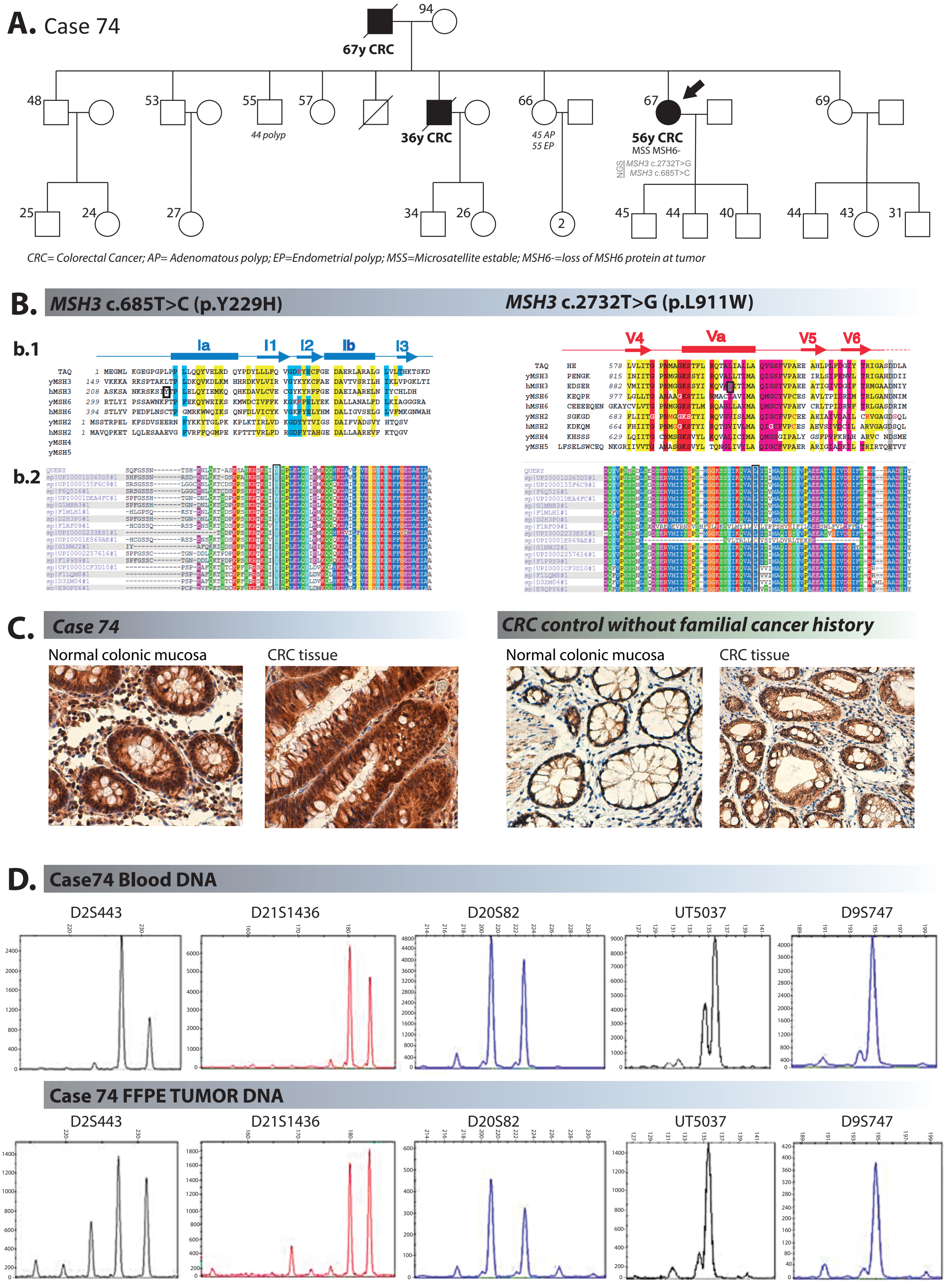

A. Case 74
67y CRC
36y CRC
56y CRC
MSS MSH6-
NGS
MSH3 c.2732T>G
MSH3 c.685T>C
44 polyp
45 AP
55 EP
CRC= Colorectal Cancer; AP= Adenomatous polyp; EP=Endometrial polyp; MSS=Microsatellite estable; MSH6-=loss of MSH6 protein at tumor
B. MSH3 c.685T>C (p.Y229H)
MSH3 c.2732T>G (p.L911W)
b.1
b.2
C. Case 74
Normal colonic mucosa
CRC tissue
CRC control without familial cancer history
Normal colonic mucosa
CRC tissue
D. Case74 Blood DNA
D2S443
D21S1436
D20S82
UT5037
D9S747
Case 74 FFPE TUMOR DNA
D2S443
D21S1436
D20S82
UT5037
D9S747
