# Supplementary material for: Comprehensive Constitutional Genetic and Epigenetic Characterization of Lynch-Like Individuals
Source: Cancers (Basel). 2020 Jul 5;12(7):1799. doi: 10.3390/cancers12071799 (PMC7408773; doi:10.3390/cancers12071799)
Supplement: Supplementary file 1 [file cancers-12-01799-s001.zip › Figure S5_Contribution of COSMIC mutational signatures.pdf]

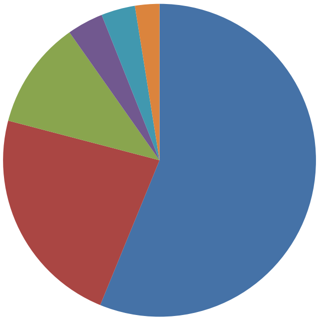

Mutational signature	Contribution (%)	Proposed etiology
Signature 6	56,19	Defective DNA mismatch repair
Signature 1	22,89	Deamination of 5-methylcytosine (present in all cancer types)
Signature 16	11,16	-
Signature 26	3,73	Defective DNA mismatch repair
Signature 12	3,51	-
Signature 7	2,52	UV exposure
