# Supplementary figures and images for: Comprehensive Constitutional Genetic and Epigenetic Characterization of Lynch-Like Individuals
Source: Cancers (Basel). 2020 Jul 5;12(7):1799. doi: 10.3390/cancers12071799 (PMC7408773; doi:10.3390/cancers12071799)

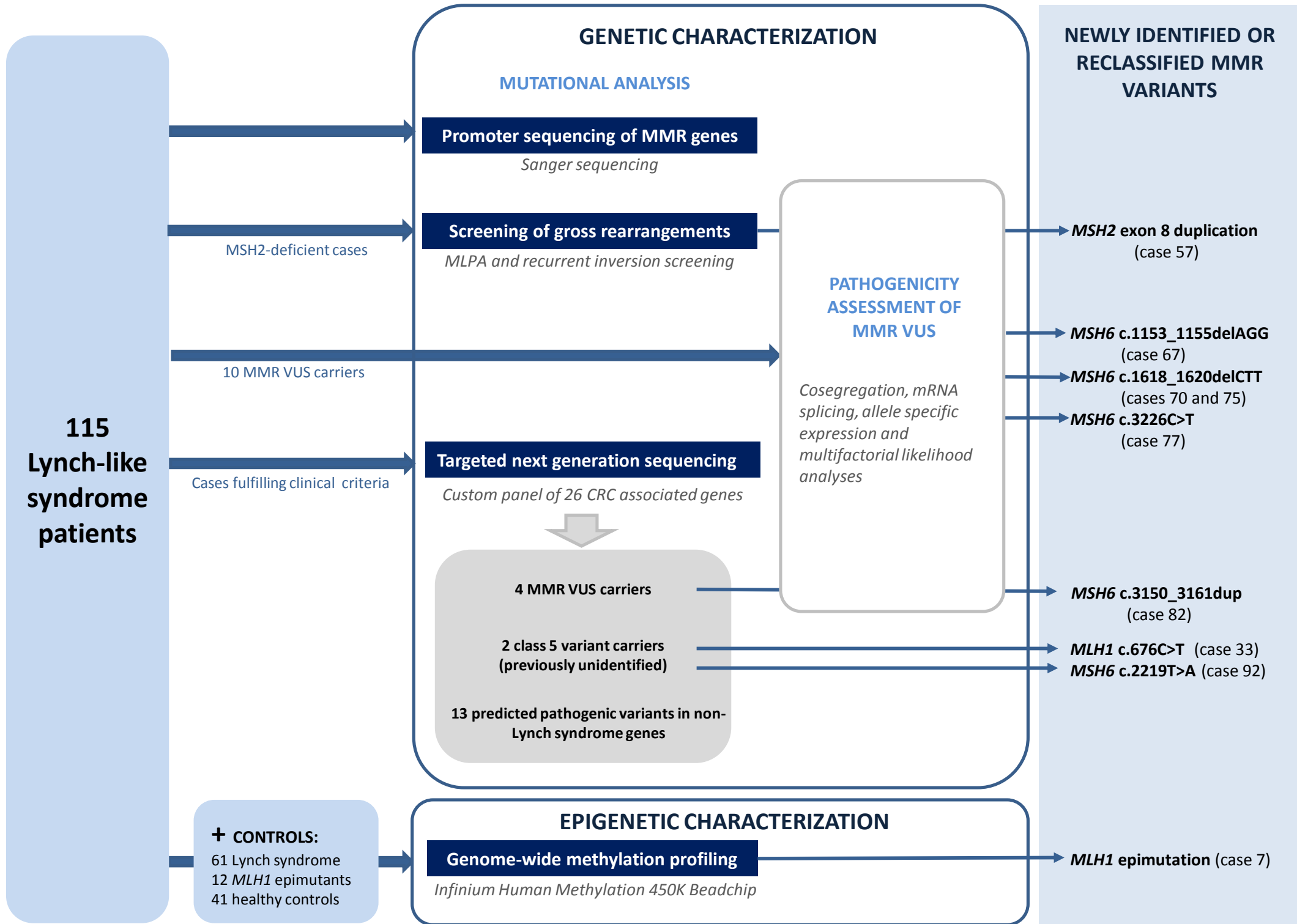

Supplement: Supplementary file 1 [file cancers-12-01799-s001.zip › Appendix Figure 1_Workflow_new.pdf]

# A

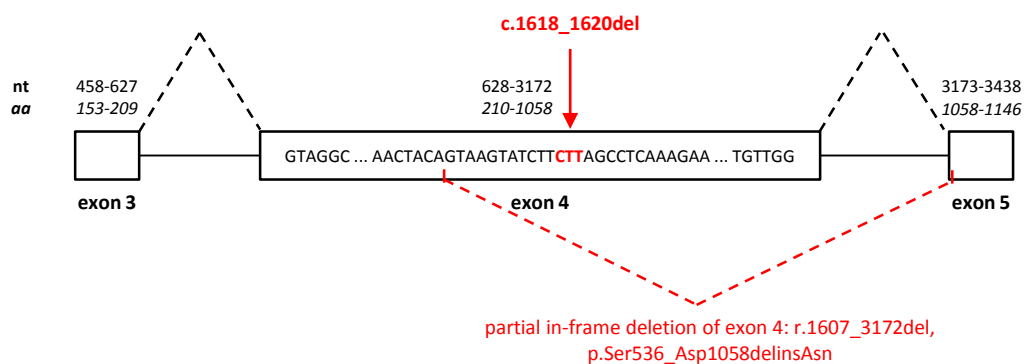

# B

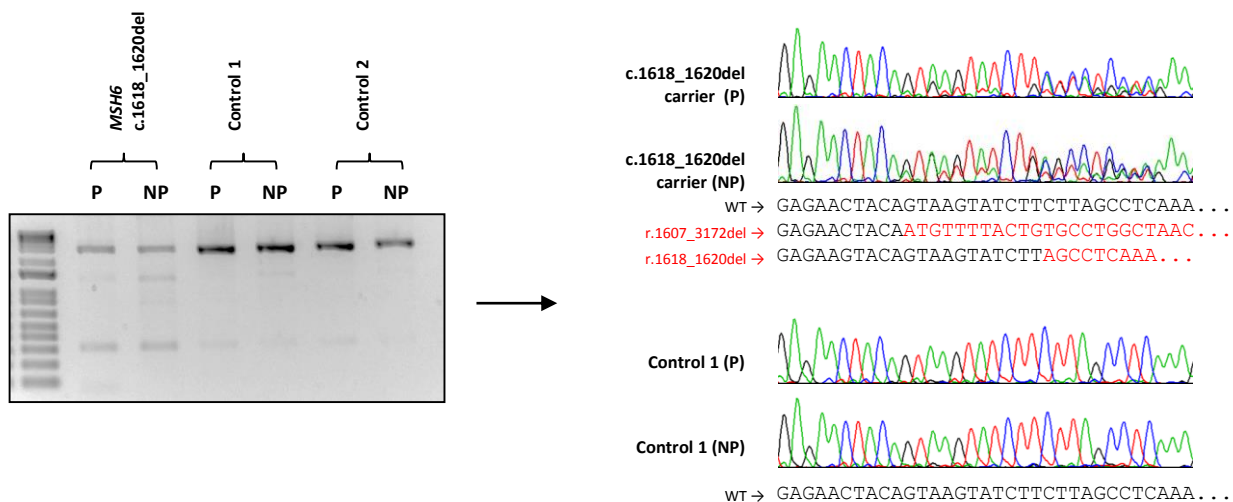

Supplement: Supplementary file 1 [file cancers-12-01799-s001.zip › Figure S1_Splicing analysis of MSH6 c1618_1620del variant.pdf]

**A. AF1** (*MSH6* c.1153\_1155delAGG)

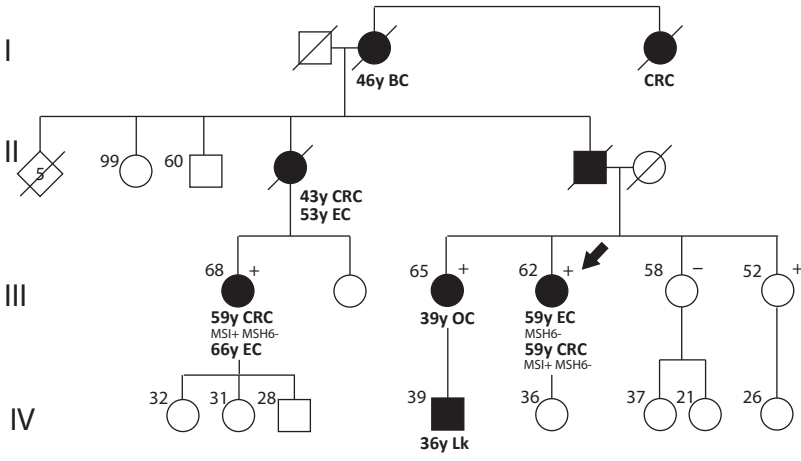

**B. AF2** (*MSH6* c.3150\_3161dup)

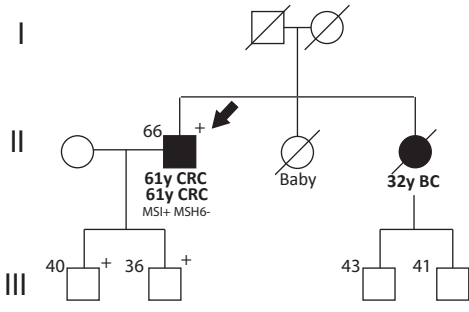

**C. AF3** (*MSH6* c.3150\_3161dup)

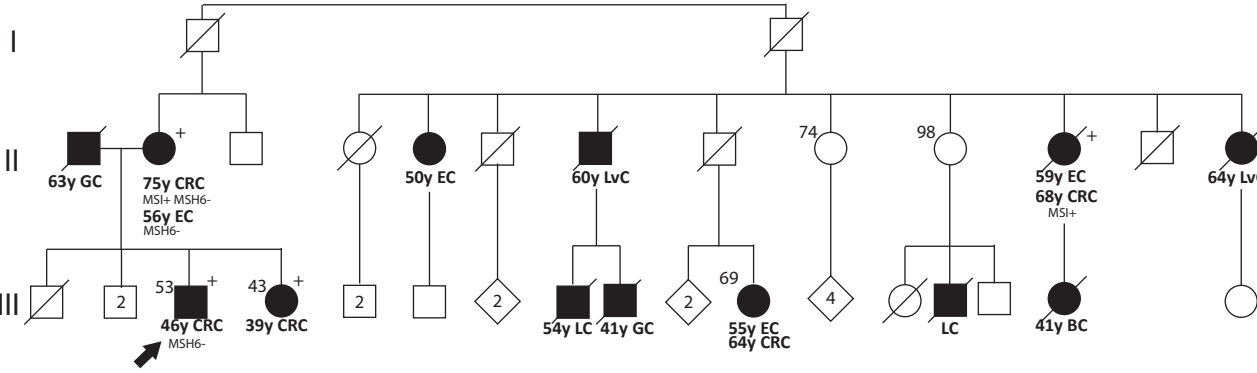

Supplement: Supplementary file 1 [file cancers-12-01799-s001.zip › Figure S2_Additional families.pdf]

# A

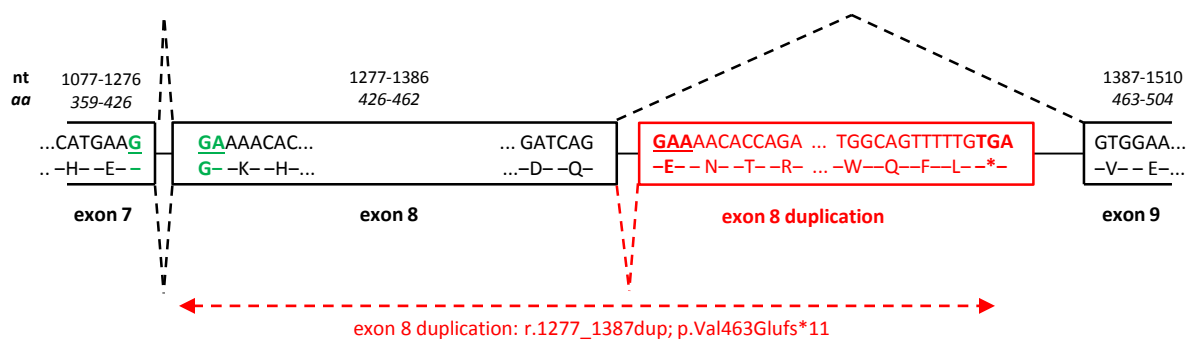

# B

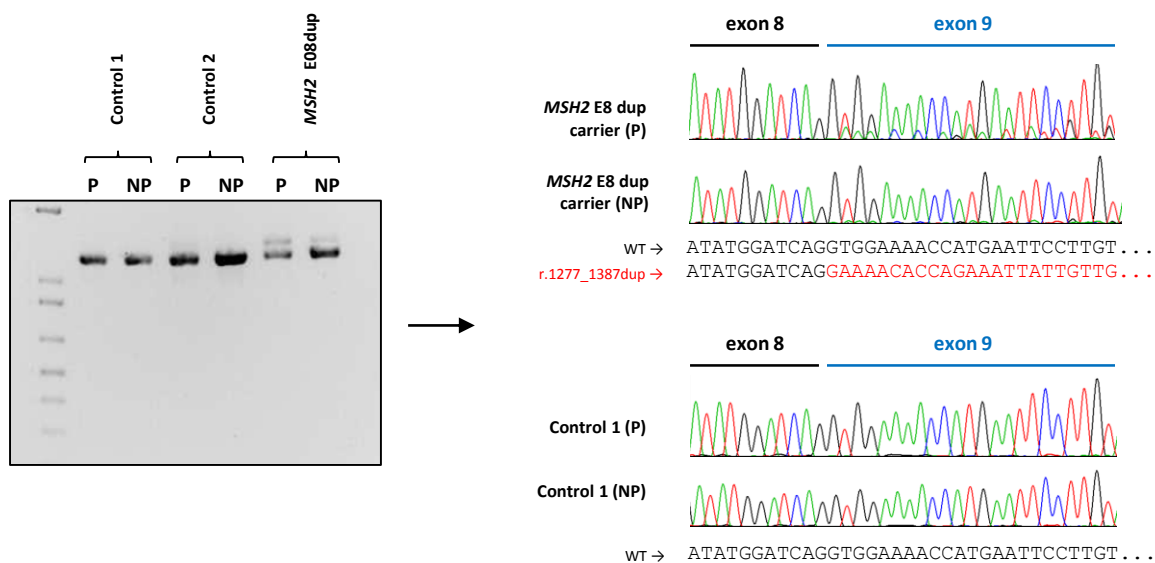

Supplement: Supplementary file 1 [file cancers-12-01799-s001.zip › Figure S3_Splicing analysis of MSH2 exon 8 duplication.pdf]

**A.**

CpG islands:

Ref.Genes:

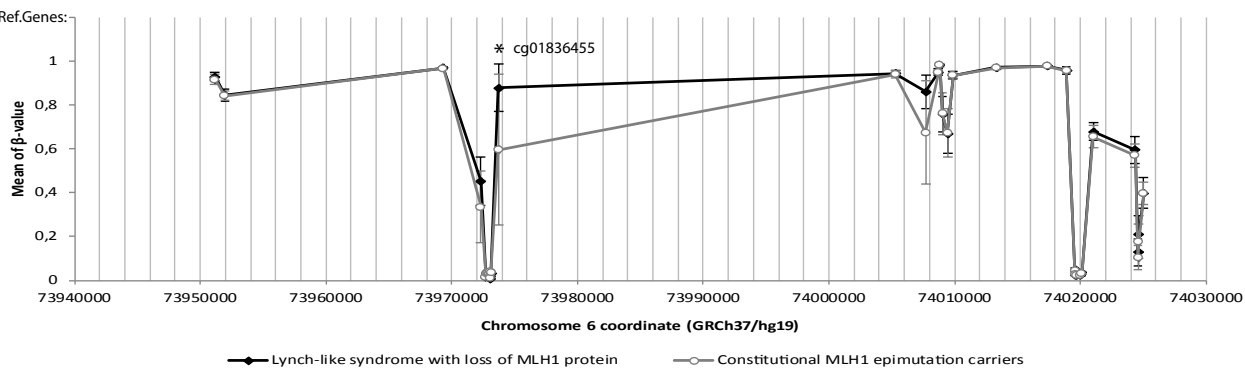**B.**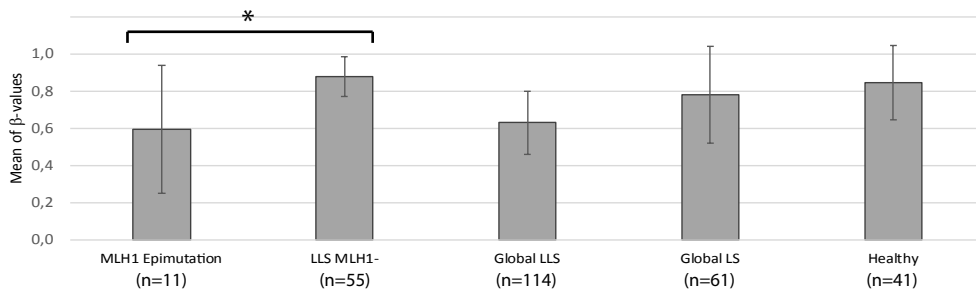

Supplement: Supplementary file 1 [file cancers-12-01799-s001.zip › Figure S6_Differentially methylated CpG site at KHDC1 gene.pdf]
